# Reversible Electrical Control of Interfacial Charge Flow across van der Waals Interfaces

**DOI:** 10.1021/acs.nanolett.2c04795

**Published:** 2023-02-17

**Authors:** Shuai Fu, Xiaoyu Jia, Aliaa S. Hassan, Heng Zhang, Wenhao Zheng, Lei Gao, Lucia Di Virgilio, Sven Krasel, David Beljonne, Klaas-Jan Tielrooij, Mischa Bonn, Hai I. Wang

**Affiliations:** † Max Planck Institute for Polymer Research, Ackermannweg 10, D-55128 Mainz, Germany; ‡ School of Physics and Key Laboratory of MEMS of the Ministry of Education, Southeast University, Nanjing 211189, China; § Laboratory for Chemistry of Novel Materials, Université de Mons, 20 Place du Parc, 7000 Mons, Belgium; ∥ Catalan Institute of Nanoscience and Nanotechnology (ICN2), BIST & CSIC, Campus UAB, Bellaterra, Barcelona 08193, Spain

**Keywords:** van der Waals heterostructures, charge transfer, photogating, electrochemical gating, operando terahertz spectroscopy

## Abstract

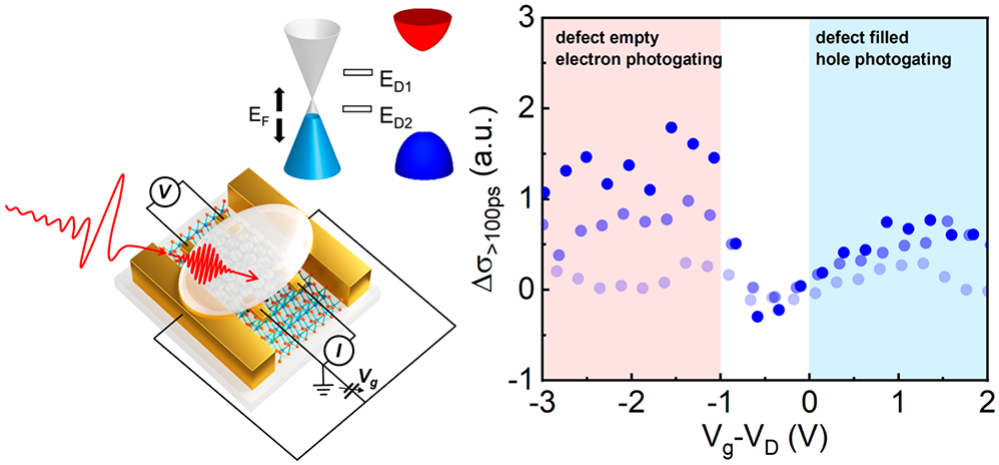

Bond-free integration of two-dimensional (2D) materials yields van der Waals (vdW) heterostructures with exotic optical and electronic properties. Manipulating the splitting and recombination of photogenerated electron–hole pairs across the vdW interface is essential for optoelectronic applications. Previous studies have unveiled the critical role of defects in trapping photogenerated charge carriers to modulate the photoconductive gain for photodetection. However, the nature and role of defects in tuning interfacial charge carrier dynamics have remained elusive. Here, we investigate the nonequilibrium charge dynamics at the graphene–WS_2_ vdW interface under electrochemical gating by operando optical-pump terahertz-probe spectroscopy. We report full control over charge separation states and thus photogating field direction by electrically tuning the defect occupancy. Our results show that electron occupancy of the two in-gap states, presumably originating from sulfur vacancies, can account for the observed rich interfacial charge transfer dynamics and electrically tunable photogating fields, providing microscopic insights for optimizing optoelectronic devices.

Vertical stacking of atomically thin materials constitutes van der Waals (vdW) heterostructures with exotic physics and synergistic properties beyond lattice-matching constraints. Such artificial material manipulation provides fertile ground for exploring fundamental physics and enabling novel optoelectronics. Among the vast gamut of vdW heterostructures, graphene–transition metal dichalcogenide (TMDC) heterostructures have received particular attention because they inherit strong light–matter interactions and high charge carrier mobility from their constituent building blocks.^1−5^ At the bond-free interface, graphene and TMDC are coupled by vdW forces.^6^ The resulting interlayer atomic interactions enable strong electronic coupling, leading to interfacial charge flow which underpins various optoelectronic applications,^7,8^ including photodetection.

As the backbone of these applications, understanding and tuning the spatial distribution of photogenerated electron–hole pairs at the vdW interface is paramount for photocurrent operation. For that, recent spectroscopic studies have investigated photoinduced interfacial processes in graphene–TMDC heterostructures using ultrafast pump–probe techniques.^9−17^ It has been reported that photoinduced charge transfer (CT) in graphene–TMDC heterostructures occurs within ∼100 fs, and the activated CT trajectories critically depend on the pump photon wavelength. While direct hole tunneling from TMDC to graphene dominates the CT mechanism following resonant or above-bandgap photoexcitation,^10,12,13,17^ photothermionic emission (PTE) governs the CT process upon sub-bandgap photoexcitation.^18,19^ In the PTE scenario, the absorbed low-energy photons efficiently heat the electron bath in graphene, resulting in a thermalized hot carrier distribution. The thermalized hot carriers with sufficiently high energy can be emitted over the Schottky barrier at the graphene–TMDC interface.^10,15,18^

Following optical excitations and CT, transferred electrons and holes reside in different materials: transient charge separation states emerge. The resulting charge separation time is critical in determining the photoconductive gain and operation bandwidth for many graphene-based optoelectronic devices such as photodetectors.^20^ In our previous work,^10^ we revealed that the charge separation time is closely related to defect states in TMDCs: electrons populating the excited states of WS_2_ are rapidly and efficiently trapped by empty defect states within ∼1 ps and subsequently stored there for more than ∼1 ns. This leads to long-lived charge separation and efficient photogating in graphene, in line with the ultrahigh photoresponsivity observed in high-performance graphene–TMDC-based photodetectors.^1^ Rational defect engineering and manipulation represents one of the key strategies to further optimize the performance of optoelectronic devices. Nonetheless, realizing the full potential of defect engineering for optoelectronics is nontrivial, as it requires concerted efforts to understand the origin of defects, develop effective methods to control their nature/density, and elucidate their impact on microscopic interfacial charge carrier dynamics. So far, limited efforts have been devoted to tackling these issues.

Here, we track and manipulate nonequilibrium charge carrier dynamics in electrochemically gated graphene–WS_2_ heterostructures by operando optical-pump terahertz (THz)-probe (OPTP) spectroscopy. Electrochemical gating effectively controls the chemical potential in graphene and, thus, the interfacial energetics for CT. More importantly, it allows modulation of the filling of electronic defect states at the interface, which strongly affects the photogating mechanism. We report the first experimental implementations of electrical control of photogating field direction (i.e., the direction of the built-in interfacial electric field induced by the CT and thus photogating effect) and efficiency in vdW heterostructures: for p-doped heterostructures with empty in-gap defect states, photoinduced electron injection or trapping into defects in WS_2_ governs the photogating mechanism, leading to an “electron photogating” effect; for n-doped heterostructures with fully filled in-gap defect states, photogenerated holes are injected into defects, resulting in a “hole photogating” scheme. Such reversal of the photogating mechanism is of relevance and importance for optoelectronic applications based on vdW heterostructures. By simulating the hot carrier distribution in the heterostructure following photoexcitation, we further provide compelling experimental evidence that the observed rich interfacial CT dynamics and electrically tunable photogating fields at graphene–WS_2_ vdW interfaces can be rationalized by electron occupancy of two distinct in-gap states, presumably originating from sulfur vacancies. Our results not only reveal fundamental interfacial photophysics across the vdW interface but also provide insights into the design of advanced (opto)electronic applications through defect engineering.

As schematically shown in Figure 1a, we fabricated a vertically stacked graphene–WS_2_ heterostructure (see details in the Supporting Information). The monolayer nature of the bare WS_2_ monolayer is manifested by two pronounced optical resonances at 2.04 and 2.43 eV (Figure S1a), corresponding to the well-established A- and B-exciton transitions in WS_2_.^21^ In addition, the Raman spectrum of the bare WS_2_ monolayer shows resonances at ∼355 and ∼419 cm^–1^ (Figure S2a), corresponding to its in-plane (E_2g_^1^) and out-of-plane (A_1g_) vibration modes, in line with previous reports.^22,23^ When brought in contact with graphene, WS_2_ exhibits red-shifted exciton resonances (Figure S1b) due to the dielectric screening effect of the graphene layer.^24^ Meanwhile, the line width of the A-exciton resonance of WS_2_ in the heterostructure broadens (Figure S1b), which we attribute to the static interlayer CT process.^25^ These optical signatures indicate good electronic coupling at the vdW interface. For the heterostructure, along with the well-preserved vibration modes of WS_2_, we obtain the expected G- and 2D-bands originating from graphene (Figure S2b). The single symmetric 2D-band confirms the monolayer nature of graphene, and the G-band position at ∼1586 cm^–1^ suggests an initial *E*_F_ of ∼0.15 eV in the graphene layer (equivalent to a free carrier density *N* of ∼1.4 × 10^12^ cm^–2^; Supplementary Section 1).

**Figure 1:**
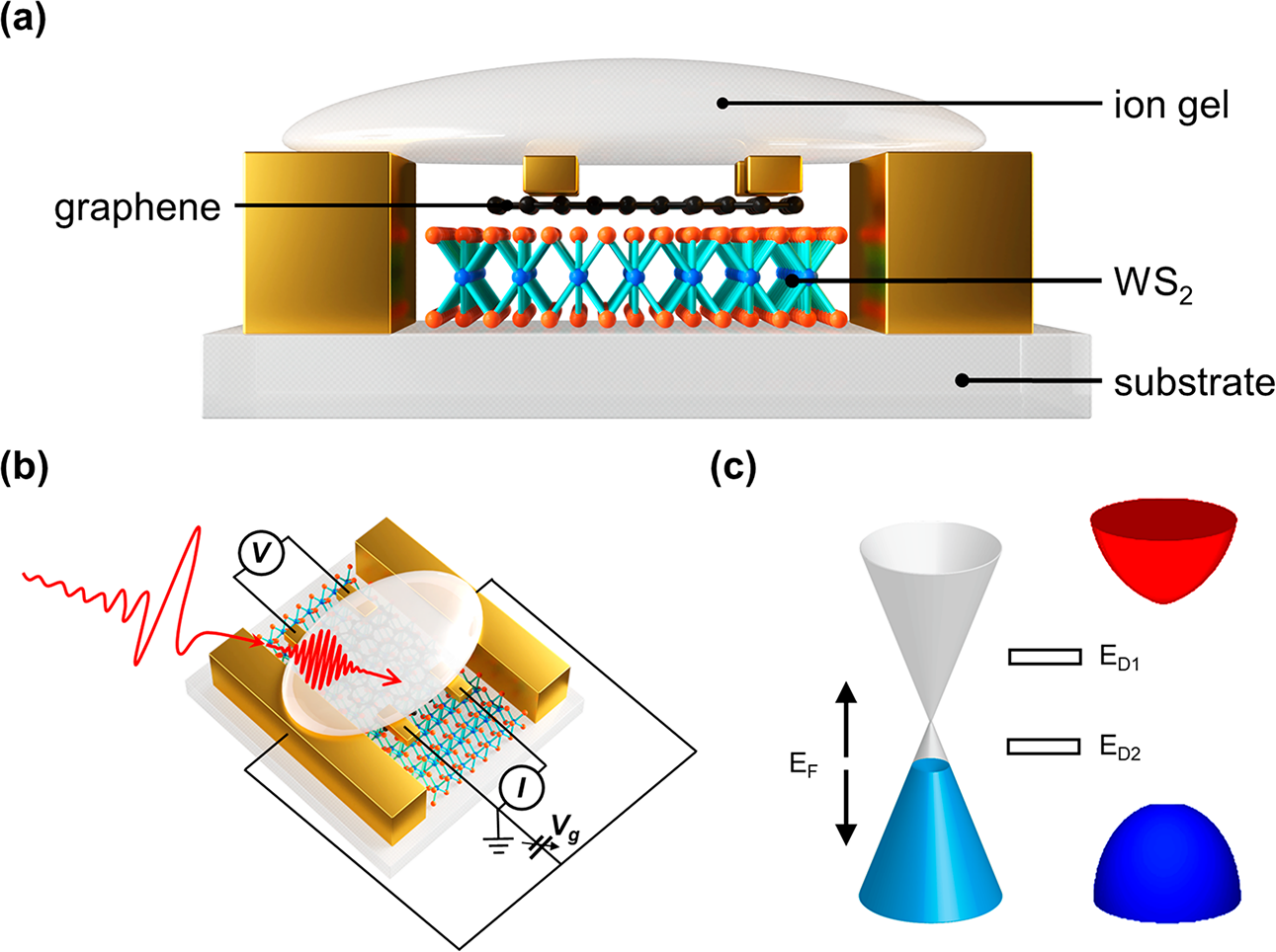
Conceptual illustration of probing and controlling interfacial charge dynamics in electrochemically gated graphene–WS_2_ vdW heterostructures by operando optical-pump THz-probe (OPTP) spectroscopy. (a) Schematic representation of a graphene–WS_2_ vdW heterostructure supported by a sapphire substrate (side view). A polymer electrolyte (LiClO_4_ dissolved in poly(ethylene oxide)) is deposited on top of the graphene layer for electrochemical gating and is transparent to light at both UV–vis and THz frequencies. Note that the gate electrodes are not in contact with the heterostructure. (b) Schematically illustration of the operando OPTP and four-point probe measurements (top view). A 1.55 eV femtosecond laser pulse selectively excites the graphene layer, and a collinearly propagating, single-cycle THz pulse probes the photoconductivity of the heterostructure as a function of pump–probe delay. The Fermi level (*E*_F_) of the heterostructure is controlled by the voltage applied between the gate and the electrode deposited on graphene. Meanwhile, the resistance of the graphene layer is measured by the four-point probe method. The measurements were performed at room temperature in a dry N_2_ environment. (c) Simplified band diagram of the heterostructure. *E*_D__1_ and *E*_D__2_ represent the energies of the two in-gap defect states involved in the photogating effect, as described in the main text.

To investigate the interfacial charge dynamics at the graphene–WS_2_ vdW interface while tuning the filling of defect states, we perform OPTP measurements on electrochemically gated graphene–WS_2_ vdW heterostructures (see details in the Supporting Information). As depicted in Figure 1b, in the OPTP measurement, a 1.55 eV ultrashort laser pulse (∼50 fs duration) selectively excites charge carriers in graphene. Subsequently, a freely propagating single-cycle THz pulse (∼1 ps duration) passes through the heterostructure. The pump-induced relative attenuation of the transmitted THz electric field (−Δ*E*/*E*) scales linearly with the photoconductivity^26^ (Δσ) and is recorded as a function of pump–probe delay time. The heterostructure used in this study is made of polycrystalline graphene and WS_2_ layers grown by chemical vapor deposition (CVD) with typical domain sizes of a few micrometers. As the diameter of the probe beam used for OPTP measurements is ∼1 mm, the measured photoconductivity dynamics represent the average response of heterostructures with mixed twist angles.

Figure 2a shows the resistance of the graphene layer as a function of gate voltage (*V*_g_ – *V*_D_). The resistance reaches a maximum value of ∼1 kΩ in the vicinity of the Dirac point and decreases in both the p-doped and n-doped regimes. Figure 2b shows the exemplary photoconductivity dynamics of the graphene–WS_2_ vdW heterostructure at different gate voltages. Notably, the photoconductivity dynamics in the first 10 ps (gray area, Figure 2b) switches its sign from positive for the low-doped regime to negative for the highly doped regimes, as also demonstrated by the evolution of the peak photoconductivity (Δσ_peak_) in Figure 2c. The sign transition of Δσ_peak_ while tuning *E*_F_ has been well reported in graphene^27−29^ and can be simply understood as follows: in intrinsic or low-doped graphene, photoexcitation leads to a substantial increase in the charge carrier density, thereby increasing the conductivity (i.e., positive Δσ_peak_). In contrast, in highly doped graphene, photoexcitation elevates the electron temperature, leading to increased momentum scattering of charge carriers and, thus, a transient reduction in conductivity (i.e., negative Δσ_peak_). Altogether, the evolution of resistance (Figure 2a) and Δσ_peak_ (Figure 2c) with gate voltage demonstrates the successful tuning of *E*_F_ in the heterostructure by electrochemical gating.

**Figure 2:**
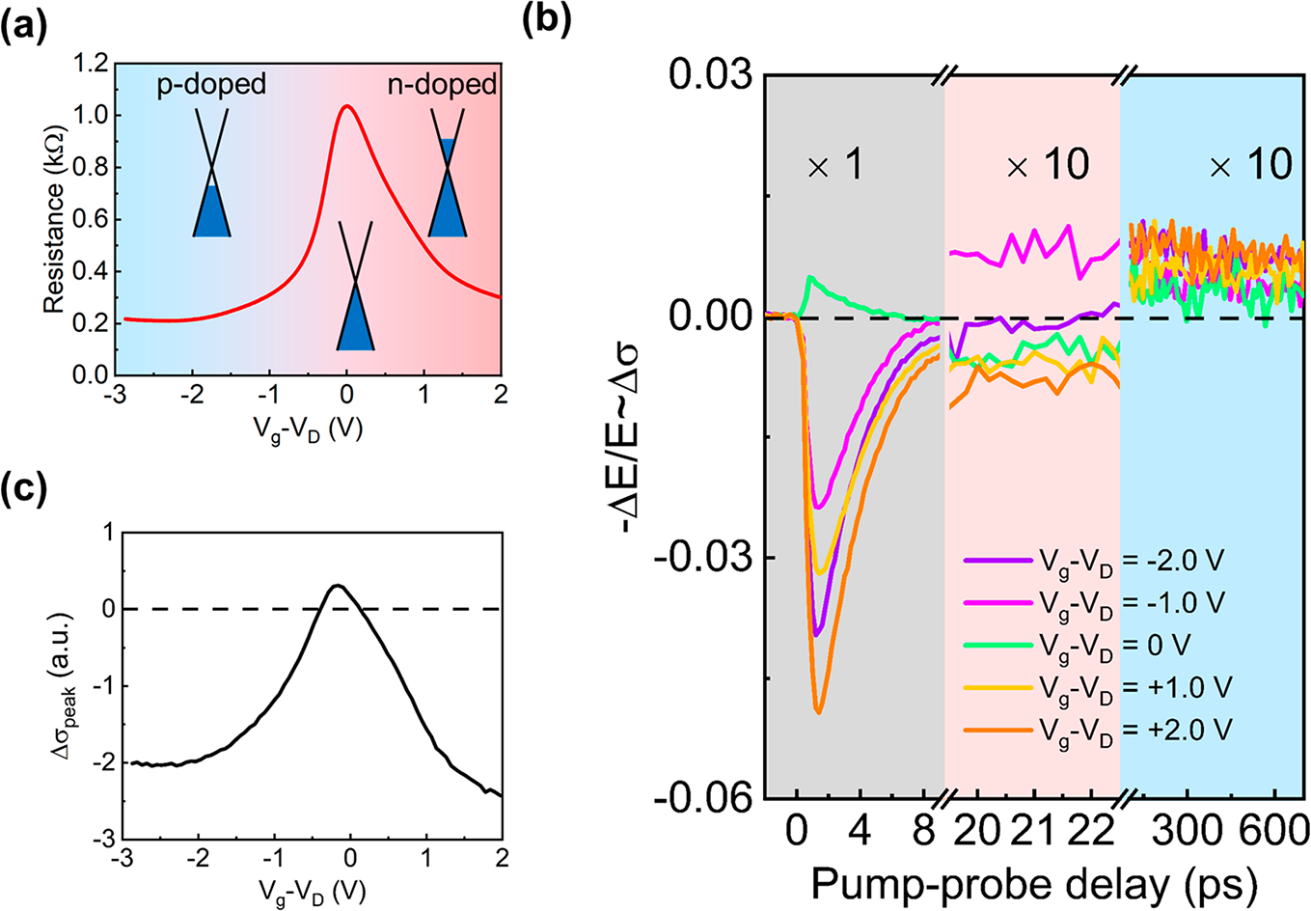
Electrically tunable interfacial charge dynamics at the graphene–WS_2_ vdW interface. (a) Electrical resistance of the graphene layer as a function of gate voltage. (b) Photoconductivity dynamics at the graphene–WS_2_ vdW interface operated at different gate voltages. The sample was photoexcited by a 1.55 eV ultrashort laser pulse with a pump fluence of 60 μJ/cm^2^. The photoconductivity dynamics are divided into three representative time windows according to their characteristics. Note that the data in the second and third time windows are multiplied by a factor of 10 for better visualization. Here *V*_g_ and *V*_D_ are the applied gate voltage and the voltage corresponding to the Dirac point of graphene, respectively. (c) Peak THz photoconductivity as a function of gate voltage.

We now focus on photoconductivity dynamics on longer time scales beyond 20 ps. Because the intrinsic hot carrier response of bare graphene is negligible within 10 ps,^27,30^ the remaining photoconductivity is directly related to the CT-induced conductivity change in graphene.^10,31−33^ As shown in Figure 2b, around 20 ps (marked in light pink), the CT-induced photoconductivity demonstrates a substantial variation with gate voltage, indicating that *E*_F_-dependent interfacial CT processes take place at the vdW interface. On the other hand, beyond 100 ps, the photoconductivity is always positive and shows a weak dependence on gate voltage (blue area, Figure 2b). For better comparison, we summarize the gating-dependent photoconductivity at around 20 ps (defined as Δσ_20ps_) and beyond 100 ps (average of data between 100 and 700 ps, defined as Δσ_*>*__100ps_) under three different pump fluences, as shown in Figures 3a,b.

**Figure 3:**
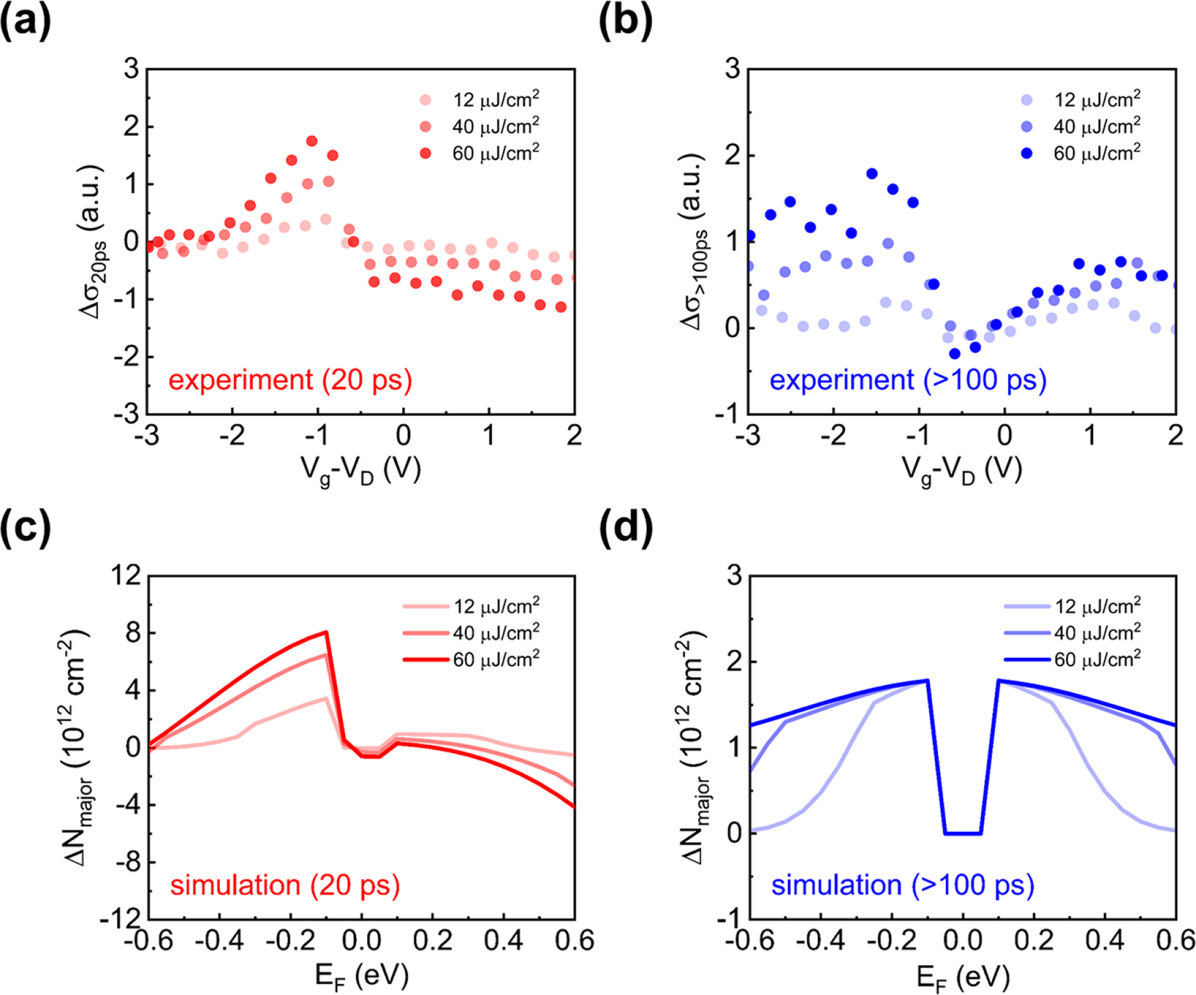
Defect-mediated CT and photogating at vdW interfaces. (a, b) Photoconductivity (a) at ∼20 ps and (b) averaged from 100 to 700 ps as a function of gate voltage under different pump fluences. (c, d) Simulated *E*_F_-dependent majority conducting charge density change in graphene based on the defect-mediated photogating scenario (c) before (at ∼20 ps) and (d) after (>100 ps) the back-transfer of charge carriers from the conduction and valence bands of WS_2_ under different pump fluences.

We observe a clear sign transition of Δσ_20ps_ upon electrochemical gating, from positive for the p-doped (referring to graphene) regime to negative for the n-doped regime. We understand the observed gating-modulated photoconductivity as CT-induced transient electron loss (i.e., the decrease of electron density) in graphene, which downshifts *E*_F_ and thus increases (decreases) the conductivity of p-doped (n-doped) graphene at all gate voltages employed. We also observe that Δσ_>100ps_ is positive regardless of the gate voltage, indicating a transition from CT-induced electron loss to gain while tuning graphene from the p- to n-doped regime. This result further indicates a reversal of the interfacial photogating field, from pointing away from graphene in the p-doped regime to pointing toward graphene in the n-doped regime. Our previous study^10^ reported that electrons trapped in empty defects in WS_2_ are responsible for photogating in p-doped heterostructures. In the following, we show that holes trapped in the electrochemically filled in-gap defects in WS_2_ are responsible for photogating in n-doped heterostructures. Furthermore, by comparing Δσ_20ps_ (Figure 3a) and Δσ_>100ps_ (Figure 3b), we find that the CT-induced photoconductivity undergoes a negative-to-positive transition in the n-doped regime. This indicates that the interfacial charge equilibrium of n-doped heterostructures following CT is time-dependent, leading to a dynamic transition from electron loss to electron gain in graphene (see detailed simulations below).

To describe the electrically tunable CT-induced photoconductivity and its evolution over time, we propose a defect-mediated CT and photogating mechanism. In this mechanism, electrochemical gating modulates the defect occupancy in WS_2_, thereby regulating interfacial CT channels and long-lived charge separation states across the interface. Here we assume that (i) CT occurs at the graphene–WS_2_ vdW interface only via PTE following sub-bandgap excitation in graphene, based on previous reports;^10,15,18,34^ (ii) the conduction band of WS_2_ and empty in-gap defect states serve as electron acceptors, while the valence bands of WS_2_ and electrochemically filled in-gap defect states are hole acceptors, whenever the interfacial energetics is favorable; and (iii) following CT, the back-transfer makes the system relax back to initial conditions and dictates the charge separation lifetime. We assume that the lifetime of charge carriers trapped in the defect states is substantially longer than that of charge carriers populated in the conduction and valence bands of WS_2_ because of their localized nature.^10^ Within this framework, we simulate thermalized hot carrier distributions in graphene at different initial *E*_F_ following photoexcitation and thermalization by taking into account *E*_F_-dependent heat capacity, energy conservation, and particle number conservation (Supplementary Section 2). A fixed portion of the thermalized hot electrons (holes) energetically distributed above (below) the corresponding acceptor states are assumed to be able to transfer across the vdW interface.

Based on the model, a critical and open question concerns the density, filling state, and quantity of defect states involved in CT (on short time scales) and photogating processes (on long, approximately nanosecond time scales). Recent scanning tunneling microscope (STM) studies^35^ and *ab initio* theoretical calculations^36^ revealed that sulfur vacancies (V_S_) in WS_2_ forms two energetically narrow unoccupied defect states inside the bandgap because of an exceptionally strong spin–orbit coupling in WS_2_. Furthermore, the presence of defect states in WS_2_ has been supported by ultrafast spectroscopic studies, where defects lead to rapid trapping in WS_2_^46^ and long-lived interfacial charge separation at the graphene–WS_2_ interface.^10^ The typical densities of these defects lie in the range of 10^11^–10^13^ cm^–2^.^35,36^ Given the operating range of the electrochemical gating method, we electrically tune only the filling of the in-gap defect states, while that of the filled defect state in the valence band of WS_2_ is beyond our reach. In our simulation, we consider three scenarios: (i) none, (ii) one, and (iii) two in-gap defect states. The defect density is realistically varied between 10^11^ and 10^13^ cm^–2^, and its (or their) energetics are fully relaxed within the bandgap of WS_2_ for simulation.

As discussed in Supplementary Sections S3 and S4, we find that models involving no or only one in-gap defect state are unable to reproduce the main features shown in Figure 3a,b. Briefly, in the absence of defect states, the conduction and valence bands of WS_2_ are the only CT channels to harvest thermalized hot electrons and hot holes, respectively, from graphene. We calculate the majority conducting carrier density change (Δ*N*_major_; electrons for n-doped graphene and holes for p-doped graphene) induced by CT in graphene using the difference between the number of thermalized hot electrons distributed above the conduction band minimum of WS_2_ and the number of thermalized hot holes distributed below the valence band maximum of WS_2_ (Supplementary Section 3). As shown in Figure S3, the pure PTE model, without considering any defect states, fails to reproduce the main features of the experimental results, in particular for Δσ_>100ps_: (i) in the n-doped regime, the absolute value of the CT-induced (negative) photoconductivity continuously increases with the upward shift of *E*_F_ because the increasing *E*_F_ makes hot electron transfer energetically more favorable than hot hole transfer; (ii) following the sub-100 ps back-CT process, the photoconductivity in the hybrid system is expected to relax back to the equilibrium state without exhibiting photogating effects (Figure S3b), which is in contradiction with the experimental results. Similarly, the PTE model involving only one in-gap defect state also fails to reproduce our experimental observations. This is because, following back-CT at ∼100 ps, Δ*N*_major_ immediately flips sign when *E*_F_ crosses the Dirac point and the defect energy (Supplementary Figures S4 and S5). On the other hand, the two-defect model illustrated in Figure 4 (as discussed below) captures all the essential gating- and time-dependent photoconductivity characteristics in Figure 3a,b: Δ*N*_major_ is zero in the undoped regime and becomes positive in the doped regimes, as shown in Figure 3c,d.

**Figure 4:**
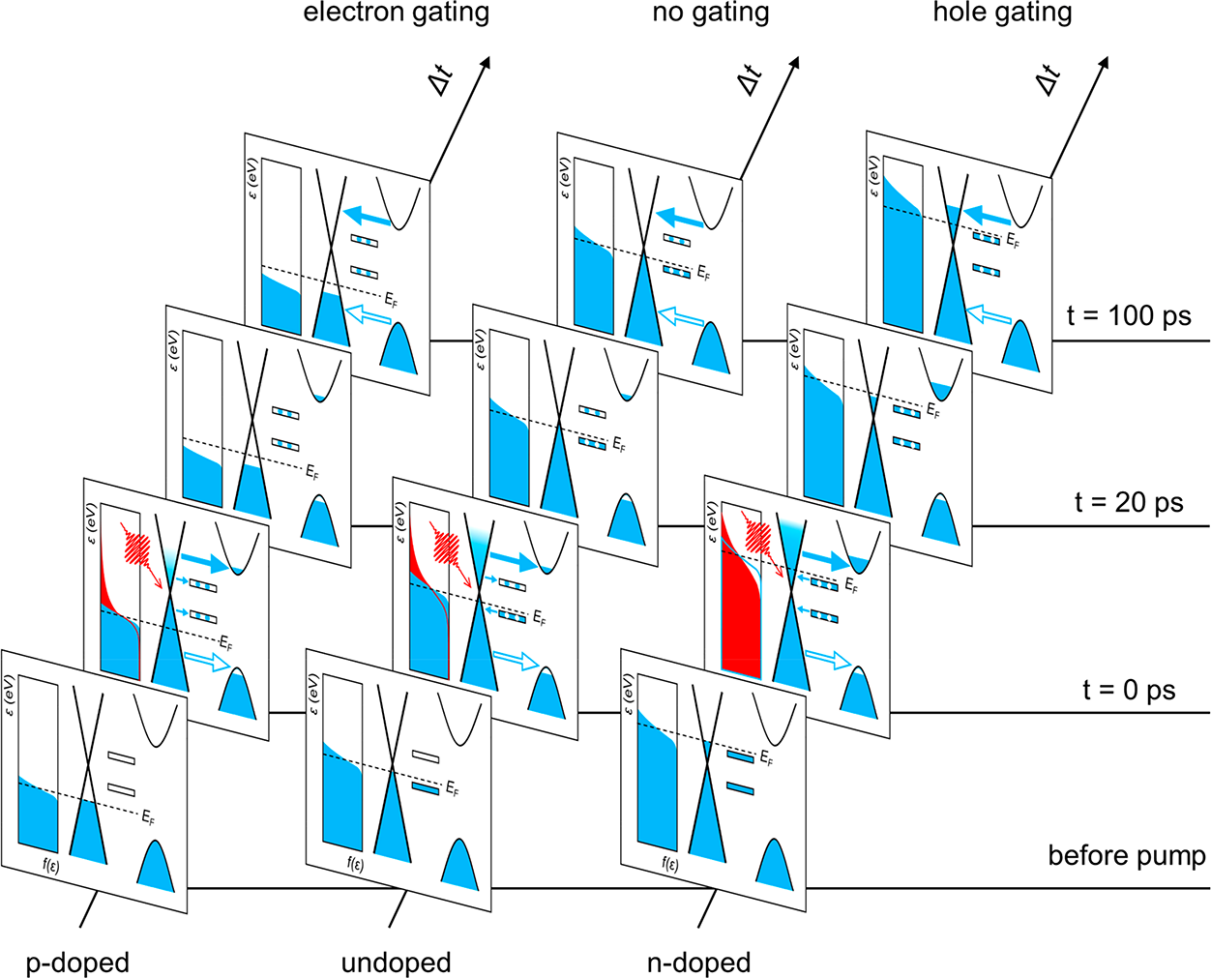
Snapshots illustrating the nonequilibrium interfacial processes that take place in p-doped, undoped, and n-doped graphene–WS_2_ vdW heterostructures following photoexcitation and thermalization.

On this basis, we further discuss the PTE model involving two in-gap defect states following the schemes shown in Figure 4. For highly p-doped heterostructures, both in-gap defect states are empty and therefore serve as electron acceptors (left panel in Figure 4). In contrast, for the highly n-doped case, the in-gap defect states are fully occupied and can trap photogenerated holes following optical excitations and CT (right panel in Figure 4). When *E*_F_ lies between these two in-gap defect states, electron trapping to the empty in-gap defect state and hole trapping to the occupied in-gap defect state can take place simultaneously, resulting in approximately null net gating charges residing in WS_2_ and negligible photogating effects (middle panel in Figure 4). For ease of description, we define the energy and density of the energetically higher defect state as *E*_D__1_ and *N*_D__1_ and those of the energetically lower defect state as *E*_D__2_ and *N*_D__2_. By numerically calculating Δ*N*_major_ at different *E*_F_ (Supplementary Section 5) following the above-mentioned schemes, we can reproduce all the main features shown in Figure 3a,b under the three different pump fluences used in the experiments, as shown in Figure 3c,d. From the simulation results, we find that on early time scales (∼20 ps), the electron loss at all gate voltages can be rationalized by the fact that PTE of thermalized hot electrons to the conduction band of WS_2_ is energetically more favorable than that of thermalized hot holes to the valence band of WS_2_. On longer time scales (>100 ps), in line with our assumption and previous reports, the nature and density of defects dictate the photogating mechanisms. As shown in Figure 3d, beyond 100 ps, following the back-transfer of electrons and holes from WS_2_, the photoinjected or trapped electrons in the empty in-gap defect states in the p-doped heterostructure lead to an “electron photogating” scheme in graphene, while trapped holes in the initially filled in-gap defect states cause a “hole photogating” mechanism. Furthermore, we notice that the effect of pump fluence on Δσ_>100ps_ depends largely on *E*_F_. In the doped regimes, Δσ_>100ps_ increases sublinearly with pump fluence: it gets gradually saturated with fluence (starting from the low-gating potential side), limited by the defect density (fixed for a given sample) used for the photogating. In the undoped regime, Δσ_>100ps_ shows no obvious pump fluence dependence and is close to zero. This is a result of the trade-off between electron loss and gain in graphene, as *E*_F_ lies between the two defect states. Our proposed model simplifies some detailed processes that may contribute to CT. For instance, we have assumed that the quantum yield of CT is *E*_F_-independent and linearly scales with Δ*N*_major_, which neglects the rate competition between CT and hot carrier relaxation in graphene (which is *E*_F_-dependent). The slightly asymmetric gating-dependent photoconductivity shown in Figure 3b may be partially attributed to such an assumption, which could be further augmented by the electron–hole mobility asymmetry in graphene.^37,38^

The results shown in Figure 3 clearly demonstrate the critical role of electrochemical gating on interfacial CT dynamics. For forward CT, graphene loses electrons in both n- and p-doped regimes, i.e., the CT direction is not modulated. On the other hand, electrochemical gating substantially modifies the long-lived charge separation states (electrons in empty in-gap defect states or holes in filled in-gap defect states) and, thus, the photogating field by controlling the defect occupancy. Although defects states can also be populated by other methods (e.g., electrical or electrochemical methods) to induce gating effects, the photogating effect reported in this study is unique because conductance modulation is triggered by light absorption and the subsequent CT process, which holds great promise for light sensing. Furthermore, in contrast to the conventional photogating mechanism in which the interfacial photogating field direction is determined by the CT direction (e.g., electron injection from graphene to WS_2_ leads to electron gating to graphene^10^), our study provides a novel scheme to electrically control the photogating field direction by tuning defect occupancy without changing the CT direction.

We now discuss the nature of the defects that govern interfacial photophysics in our study. While other possibilities cannot be completely ruled out, we show compelling evidence that considering only the energetics and density of in-gap defect states generated by sulfur vacancies (V_S_) can explain the electrically tunable photoconductivity dynamics shown in Figures 2 and 3. As experimentally revealed by STM and theoretically calculated by *ab initio* theory, V_S_ in WS_2_ has a typical density of 10^11^–10^13^ cm^–2^ and forms two low-lying in-gap defect states,^35,36^ which perfectly match the inferred in-gap defect density of ∼10^12^ cm^–2^ and state quantity of 2. Furthermore, on the basis of simulations, we deduce that the energy positions of the two defect states should be relatively symmetric with respect to the Dirac point of graphene. The inferred energetics are in good agreement with recent STM studies^35^ of graphene–WS_2_ vdW heterostructures.

Sulfur vacancies are known to be prevalent in both exfoliated and CVD TMDCs due to their low formation energy.^39,40^ Recent advances in defect engineering enable fine-tuning of defect density and species in TMDCs,^41−44^ which is relevant for optimizing photogating and thus photodetection applications of graphene–TMDC heterostructures. For instance, V_S_ can be either generated in a controlled fashion by electron beam irradiation^45^ or chemically repaired by rational use of superacids and/or sulfur-containing agents.^46,47^ The ability to precisely control the initial defect type/density and implement electrical control of *E*_F_ and defect filling in graphene–TMDC heterostructures provides a new degree of freedom for manipulating interfacial dynamics at the vdW interface and developing high-performance optoelectronic devices. On the other hand, rational passivation of V_S_ and fabrication of undoped heterostructures can suppress defect-induced long-lived photogating at the interface, which is relevant for designing graphene–TMDC-based modulators with fast modulation speed and wide bandwidth.

In summary, we investigate *in situ* the interfacial charge dynamics in graphene–WS_2_ vdW heterostructures under electrochemical gating. The *E*_F_ modulation reversibly controls the defect occupancy in WS_2_ and the hot carrier distribution in graphene, allowing precise tuning of the direction and efficiency of the photogating field at the vdW interface. This work provides a fundamental understanding and control of the complex interfacial charge dynamics in vdW heterostructures, opening up new possibilities for developing optoelectronic devices via defect and doping engineering.

## Supporting Information Available

Additional information for sample fabrication; operando optical-pump THz-probe spectroscopy; UV–vis and Raman characterizations, and simulation details of thermalized hot carrier distribution in graphene and photothermionic emission models involving no defect, one defect, and two defects (PDF)
