# Supplementary material for: Reversible Electrical Control of Interfacial Charge Flow across van der Waals Interfaces
Source: Nano Lett. 2023 Feb 17;23(5):1850–7. doi: 10.1021/acs.nanolett.2c04795 (PMC9999450; doi:10.1021/acs.nanolett.2c04795)
Supplement: Supplementary file 1 — nl2c04795_si_001.pdf [file nl2c04795_si_001.pdf]

Supporting information for

Reversible Electrical Control of Interfacial Charge Flow Across van der Waals Interfaces

Shuai Fu[1], Xiaoyu Jia[1], Aliaa.S.Hassan[1#], Heng Zhang[1], Wenhao Zheng[1], Lei Gao[1,2], Lucia Di Virgilio[1], Sven Krasel[1], David Beljonne[3], Klaas-Jan Tielrooij[4], Mischa Bonn[1], and Hai I. Wang[1]*

[1]*Max Planck Institute for Polymer Research, Ackermannweg 10, D-55128 Mainz, Germany*

[2]*School of Physics and Key Laboratory of MEMS of the Ministry of Education, Southeast University, Nanjing 211189, China*

[3]*Laboratory for Chemistry of Novel Materials, Université de Mons, 20 Place du Parc, 7000 Mons, Belgium*

[4]*Catalan Institute of Nanoscience and Nanotechnology (ICN2), BIST & CSIC, Campus UAB, Bellaterra, Barcelona 08193, Spain*

*Corresponding author: wanghai@mpip-mainz.mpg.de

# Current address: *Chair of Biogenic Functional Materials, Technical University of Munich, Schulgasse 22, D-94315 Straubing, Germany*

Contents

I. Methods

II. Supplementary sections

Section S1 Estimation of initial Fermi energy and carrier density in graphene

Section S2 Thermalized hot carrier distribution in graphene

Section S3 Pure photo-thermionic emission (PTE) model

Section S4 PTE model involving one defect state

Section S5 PTE model involving two defect states

III. Supplementary figures

Figure S1 UV-vis absorption spectra of WS_2 monolayer and graphene-WS_2 heterostructure

Figure S2 Raman spectra of WS_2 monolayer and graphene-WS_2 heterostructure

Figure S3 Majority conducting carrier density change in graphene based on the pure PTE model

Figure S4 Majority conducting carrier density change in graphene based on the PTE model involving one defect state with a fixed defect density

Figure S5 Majority conducting carrier density change in graphene based on the PTE model involving one defect state with a fixed defect energy

Figure S6 An optical image of the as-prepared electrochemically gated graphene-WS_2 heterostructure

I. Methods

(A) Fabrication of graphene-WS_2 van der Waals heterostructures

CVD-grown WS_2 monolayers supported by sapphire substrates were commercially available and purchased from SixCarbon Technology (Shenzhen). Afterwards, CVD-grown graphene was transferred onto the WS_2 monolayer according to the following procedure. First, cellulose acetate butyrate (CAB) was dissolved in ethyl acetate (30 mg/mL) and spin-coated on graphene supported by copper foil at 4000 rpm. After baked at 180 °C for 3 min, the copper foil was etched with 3 g/100 ml aqueous ammonium persulfate solution. Subsequently, the CAB-coated graphene was rinsed 5 times with Milli-Q water to remove etchant residues, and then transferred onto the WS_2 monolayer to form the graphene-WS_2 vdW heterostructure. After 2 days of natural drying, CAB was removed by soaking with acetone (12 h) and isopropanol (1 h). Ti/Au (5/50 nm) was deposited on the heterostructure through a shadow mask to form source, drain, and gate electrodes. $LiClO_4$ dissolved in polyethylene oxide was deposited on the heterostructure and used as a high-capacitance gate dielectric to perform electrochemical gating. The sample preparation process was performed in a dust-free environment to avoid potential contaminants.

(B) Operando optical-pump THz-probe spectroscopy

Operando optical-pump THz-probe (OPTP) spectroscopy was employed to monitor in situ the photoconductivity of the heterostructure under device operation. The OPTP spectroscopy was driven by a regenerative amplified and mode-locked Ti:sapphire system, which generated femtosecond laser pulses with a central energy of 1.55 eV, a pulse duration of ~50 fs, and a repeating frequency of 1 kHz. Optical excitation directly utilized the generated 1.55 eV laser pulses. Single-cycle THz pulses of ~1 ps duration were generated via optical rectification by pumping a (110) ZnTe crystal with 1.55 eV ultrafast laser pulses. The resulting THz pulses were collimated and focused onto the sample by a pair of off-axis parabolic mirrors. The transmitted THz pulses were then re-collimated and focused onto the second (110) ZnTe crystal to map out the time-

varying electric field by free-space electro-optic sampling. The measurements were performed at room temperature under a dry N_2 atmosphere. The resistance of the graphene layer was monitored simultaneously by the four-point probe method during OPTP measurements.

II. Supplementary sections

Section S1 Estimation of initial Fermi energy and carrier density in graphene

We estimate E_F in graphene from its G-band frequency (w_G), following equation (S1).[1]

$$w_G - 1580cm^{-1} = (42cm^{-1}eV)|E_F| \quad (S1)$$

E_F is found to be ~0.15 eV with respect to the Dirac point. On this basis, we further calculate the carrier density (N) in graphene, following equation (S2).[2]

$$|E_F| = \hbar v_F(\pi|N|)^{1/2} \quad (S2)$$

where $\hbar$ is the reduced Planck constant and v_F is the Fermi velocity. The calculated N is ~1.4×10^{12} cm^{-2}.

Section S2 Thermalized hot carrier distribution in graphene

We simulate the thermalized hot carrier distribution in graphene after photoexcitation by considering energy conservation and particle number conservation.[3] For energy conservation, the total energy of the graphene electronic system before photoexcitation $Q_0(T_L)$ contains the energies of electrons and holes, and can be written as equation (S3).

$$Q_0(T_L) = \int_0^\infty \varepsilon v(\varepsilon) f_e(E_F,T_L,\varepsilon) d\varepsilon + \int_0^\infty \varepsilon v(\varepsilon) f_h(E_F,T_L,\varepsilon) d\varepsilon \quad (S3)$$

where T_L is the lattice temperature, ε is the energy, $v(\varepsilon)$ is the density of states (DOS) at a given energy ε, $f_e(E_F,T_L,\varepsilon)$ is the Fermi-Dirac distribution for electrons, and $f_h(E_F,T_L,\varepsilon)d\varepsilon$ is the Fermi-Dirac distribution for holes. For simplicity, we assume T_L is 298 K before photoexcitation, and approximate the chemical potential in graphene at room temperature as E_F. After photoexcitation, the absorbed photon energy (ΔQ) is converted into electronic heat, increasing the effective temperature (T_e) of the electron bath in graphene. According to energy conservation, the total energy of the graphene electronic system after photoexcitation $Q_{total}(T_e)$ can be written as equation (S4).

$$Q_{total}(T_e) = Q_0(T_L) + \Delta Q = \int_0^\infty \varepsilon v(\varepsilon) f_e(\varphi,T_e,\varepsilon) d\varepsilon + \int_0^\infty \varepsilon v(\varepsilon) f_h(\varphi,T_e,\varepsilon) d\varepsilon \quad (S4)$$

ΔQ can be expressed as equation (S5).

$$\Delta Q = \gamma \cdot \varphi_{heat} \cdot Q_{in} \quad (S5)$$

where γ = 2.3%[4] is the optical absorption of graphene at the pump wavelength used (i.e., 800 nm), Q_{in} represents the energy of the incident laser pulse, and φ_{heat} stands for the heat efficiency described in reference[5] and is assumed to be 70 % here, and φ is the chemical potential after photoexcitation.

For particle number conservation, the total carrier density (n) in graphene is a constant defined by the initial chemical potential regardless of photoexcitation, and can be written as equation (S6).

$$n = \int_0^{\infty} \nu(\varepsilon)[f_e(\varphi, T_e, \varepsilon) - f_h(\varphi, T_e, \varepsilon)]d\varepsilon = \int_0^{\infty} \nu(\varepsilon)[f_e(E_F, T_L, \varepsilon) - f_h(E_F, T_L, \varepsilon)]d\varepsilon \quad (S6)$$

Based on energy conservation and particle number conservation, we can numerically calculate the changes in the effective temperature of the electron bath ($T_L \rightarrow T_e$) and chemical potential ($E_F \rightarrow \varphi$) in graphene caused by an incident laser pulse with energy input Q_{in}, thereby obtaining the thermalized hot electron distribution and hot hole distribution after photoexcitation.

Section S3 Pure photo-thermionic emission (PTE) model

Based on early studies[6–8], we set the band alignment of the graphene-WS_2 vdW heterostructure as follows: the conduction (E_{CB}) and valence (E_{VB}) bands of WS_2 are located at 0.9 eV above and 1.4 eV below the Dirac point, respectively. In the pure PTE model without considering any defect states, the conduction band of WS_2 can harvest thermalized hot electrons with energy higher than E_{CB} and the valence band of WS_2 can harvest thermalized hot holes with energy higher than E_{VB}. At given E_F and Q_{in}, the net electron loss (ΔN_e) in graphene due to PTE can be obtained by calculating the difference between the number of thermalized hot electrons distributed above E_{CB} and the number of thermalized hot holes distributed below E_{VB}, following equation (S7).

$$\Delta N_e = \int_{E_{CB}}^{\infty} v(\varepsilon) f_e(\varphi, T_e, \varepsilon) d\varepsilon - \int_{-\infty}^{E_{VB}} v(\varepsilon) f_h(\varphi, T_e, \varepsilon) d\varepsilon \quad \text{(S7)}$$

Note that we use the majority conducting carrier density change ΔN_{major} to describe the charge carrier gain or loss in graphene, as its sign can be easily linked to the photoconductivity$\Delta\sigma$. We define $\Delta N_{major} = \Delta N_e$ in n-doped graphene and $\Delta N_{major} = -\Delta N_e = \Delta N_h$ in p-doped graphene, respectively.

Section S4 PTE model involving one defect state

In this section, we consider one in-gap defect state with either a fixed density or fixed energy, and discuss the PTE model based on the occupancy of the in-gap defect state. We define the energy and density of this defect state as E_D and N_D, respectively. When E_F is energetically lower than E_D, this defect state is unoccupied and behaves as electron acceptor. In this case, ΔN_e can be calculated by equation (S8), and the defect-assisted contribution ΔN_{e_D} can be written as equation (S9).

$$\Delta N_e = \int_{E_{CB}}^{\infty} v(\varepsilon) f_e(\varphi, T_e, \varepsilon) d\varepsilon + \int_{E_D}^{E_{CB}} v(\varepsilon) f_e(\varphi, T_e, \varepsilon) d\varepsilon - \int_{-\infty}^{E_{VB}} v(\varepsilon) f_h(\varphi, T_e, \varepsilon) d\varepsilon \quad \text{(S8)}$$

$$\Delta N_{e\_D} = \int_{E_D}^{E_{CB}} v(\varepsilon) f_e(\varphi, T_e, \varepsilon) d\varepsilon \quad \text{(S9)}$$

When E_F is energetically higher than E_D, this defect state is occupied and acts as hole acceptor channel. In this case, ΔN_e can be calculated by equation (S10), and the defect-assisted contribution ΔN_{e_D} can be written as equation (S11).

$$\Delta N_e = \int_{E_{CB}}^{\infty} v(\varepsilon) f_e(\varphi, T_e, \varepsilon) d\varepsilon - \int_{E_{VB}}^{E_D} v(\varepsilon) f_h(\varphi, T_e, \varepsilon) d\varepsilon - \int_{-\infty}^{E_{VB}} v(\varepsilon) f_h(\varphi, T_e, \varepsilon) d\varepsilon \quad \text{(S10)}$$

$$\Delta N_{e\_D} = -\int_{E_{VB}}^{E_D} v(\varepsilon) f_h(\varphi, T_e, \varepsilon) d\varepsilon \quad \text{(S11)}$$

Note that we set the upper bounds of $\int_{E_D}^{E_{CB}} v(\varepsilon) f_e(\varphi, T_e, \varepsilon) d\varepsilon$ and $\int_{E_{VB}}^{E_D} v(\varepsilon) f_h(\varphi, T_e, \varepsilon) d\varepsilon$ to be N_D. Similar to Section S3, we define $\Delta N_{major} = \Delta N_e$ in n-doped graphene and $\Delta N_{major} = -\Delta N_e = \Delta N_h$ in p-doped graphene, respectively.

Section S5 PTE model involving two defect states

In this section, we consider the presence of two in-gap defect states and discuss the PTE model according to the occupancy of the two in-gap defect states. Inspired by early studies[9], we set the energetically higher defect state (E_{D1}) and the energetically lower defect state (E_{D2}) of WS_2 are symmetrically distributed around the Dirac point with an energy splitting of ~0.2 eV. The density of these two defect states (N_{D1} and N_{D2}) is set to $N_{D1} = N_{D2} = 5\times10^{11}$ cm^{-2}.[10,11]

In the p-doped heterostructure where E_F is energetically lower than E_{D2}, these two defect states are unoccupied and can be seen as electron acceptors. In this case, ΔN_e can be calculated by equation (S12), and the defect-assisted contribution ΔN_{e_D} can be written as equation (S13).

$$\Delta N_e = \int_{E_{CB}}^{\infty} v(\varepsilon) f_e(\varphi, T_e, \varepsilon) d\varepsilon + \int_{E_{D1}}^{E_{CB}} v(\varepsilon) f_e(\varphi, T_e, \varepsilon) d\varepsilon + \int_{E_{D2}}^{E_{D1}} v(\varepsilon) f_e(\varphi, T_e, \varepsilon) d\varepsilon - \int_{-\infty}^{E_{VB}} v(\varepsilon) f_h(\varphi, T_e, \varepsilon) d\varepsilon \quad \text{(S12)}$$

$$\Delta N_{e\_D} = \int_{E_{D1}}^{E_{CB}} v(\varepsilon) f_e(\varphi, T_e, \varepsilon) d\varepsilon + \int_{E_{D2}}^{E_{D1}} v(\varepsilon) f_e(\varphi, T_e, \varepsilon) d\varepsilon \quad (S13)$$

In the undoped heterostructure where E_F is located between E_{D1} and E_{D2}, E_{D1} remains unoccupied and acts as electron acceptor, whereas E_{D2} is electrically passivated and serves as hole acceptor. In this case, ΔN_e can be calculated by equation (S14), and the defect-assisted contribution ΔN_{e_D} can be written as equation (S15).

$$\Delta N_e = \int_{E_{CB}}^{\infty} v(\varepsilon) f_e(\varphi, T_e, \varepsilon) d\varepsilon + \int_{E_{D1}}^{E_{CB}} v(\varepsilon) f_e(\varphi, T_e, \varepsilon) d\varepsilon - \int_{E_{D2}}^{E_{VB}} v(\varepsilon) f_h(\varphi, T_e, \varepsilon) d\varepsilon - \int_{-\infty}^{E_{VB}} v(\varepsilon) f_h(\varphi, T_e, \varepsilon) d\varepsilon \quad \text{(S14)}$$

$$\Delta N_{e\_D} = \int_{E_{D1}}^{E_{CB}} v(\varepsilon) f_e(\varphi, T_e, \varepsilon) d\varepsilon - \int_{E_{D2}}^{E_{VB}} v(\varepsilon) f_h(\varphi, T_e, \varepsilon) d\varepsilon \quad \text{(S15)}$$

In the n-doped heterostructure where E_F is located above E_{D1}, both E_{D1} and E_{D2} are electrically passivated and serves as hole acceptors. In this case, ΔN_e can be calculated by equation (S16), and the defect-assisted contribution ΔN_{e_D} can be written as equation (S17).

$$\Delta N_e = \int_{E_{CB}}^{\infty} v(\varepsilon) f_e(\varphi, T_e, \varepsilon) d\varepsilon - \int_{E_{D2}}^{E_{D1}} v(\varepsilon) f_h(\varphi, T_e, \varepsilon) d\varepsilon - \int_{E_{VB}}^{E_{D2}} v(\varepsilon) f_h(\varphi, T_e, \varepsilon) d\varepsilon - \int_{-\infty}^{E_{VB}} v(\varepsilon) f_h(\varphi, T_e, \varepsilon) d\varepsilon \quad \text{(S16)}$$

$$\Delta N_{e\_D} = -\int_{E_{D2}}^{E_{D1}} v(\varepsilon) f_h(\varphi, T_e, \varepsilon) d\varepsilon - \int_{E_{VB}}^{E_{D2}} v(\varepsilon) f_h(\varphi, T_e, \varepsilon) d\varepsilon \quad \text{(S17)}$$

Note that we set the upper bounds of $\int_{E_{D1}}^{E_{CB}} v(\varepsilon) f_e(\varphi, T_e, \varepsilon) d\varepsilon$, $\int_{E_{D2}}^{E_{D1}} v(\varepsilon) f_e(\varphi, T_e, \varepsilon) d\varepsilon$, $-\int_{E_{D2}}^{E_{VB}} v(\varepsilon) f_h(\varphi, T_e, \varepsilon) d\varepsilon$, and $-\int_{E_{VB}}^{E_{D2}} v(\varepsilon) f_h(\varphi, T_e, \varepsilon)$ to be the defect density. Similar to Section S3, we define $\Delta N_{major} = \Delta N_e$ in n-doped graphene and $\Delta N_{major} = -\Delta N_e = \Delta N_h$ in p-doped graphene, respectively.

III. Supplementary figures

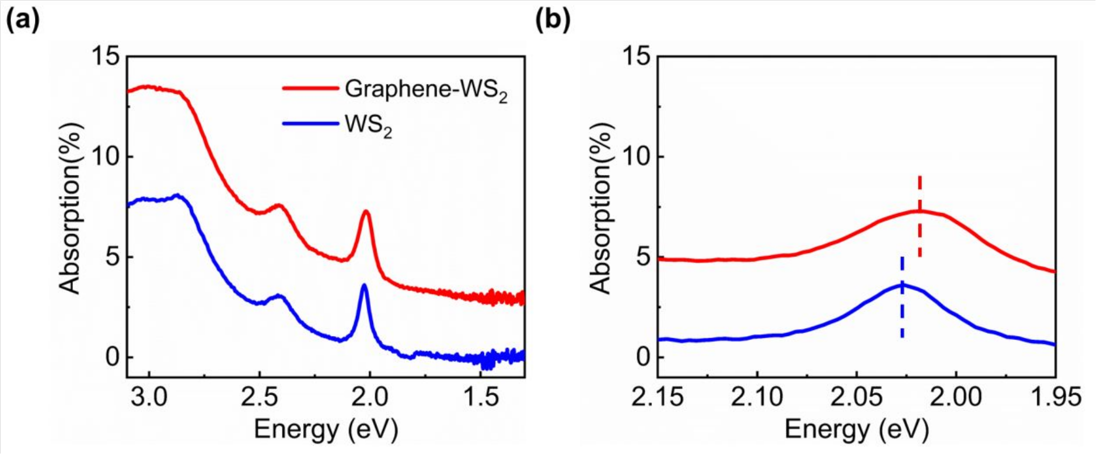

Figure S1 (a) UV-vis absorption spectra of WS_2 monolayer and graphene-WS_2 heterostructure. (b) A magnified image of A-exciton absorption.

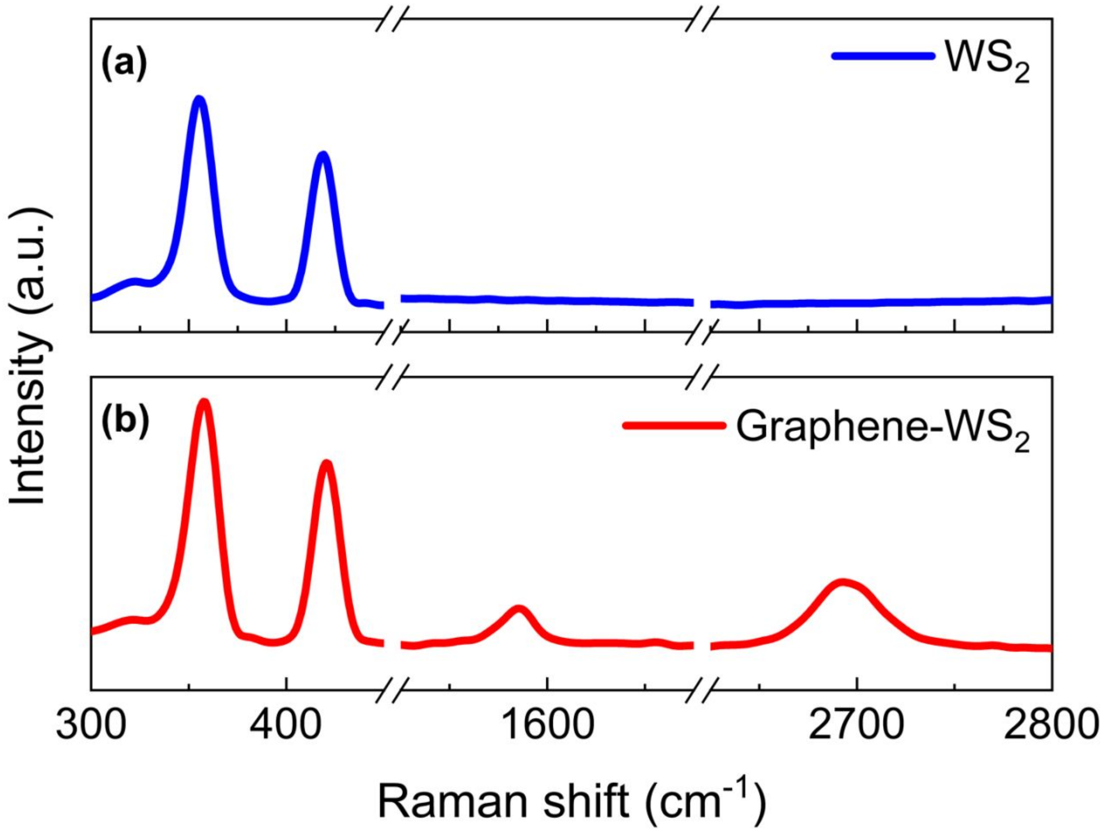

Figure S2 Raman spectra of (a) WS_2 monolayer and (b) graphene-WS_2 heterostructure.

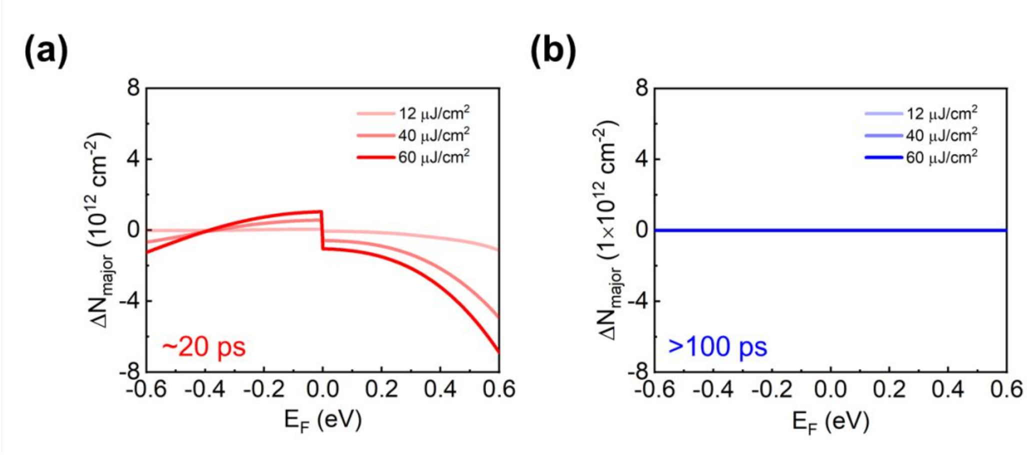

Figure S3 Majority carrier density change in graphene based on the pure PTE model (a) before (at ~20 ps) and (b) after (>100 ps) the back transfer of charge carriers from the conduction and valence bands of WS_2 under different pump fluences. Note that in Figure. S3b, ΔN_{major} for different pump fluences coincide at 0.

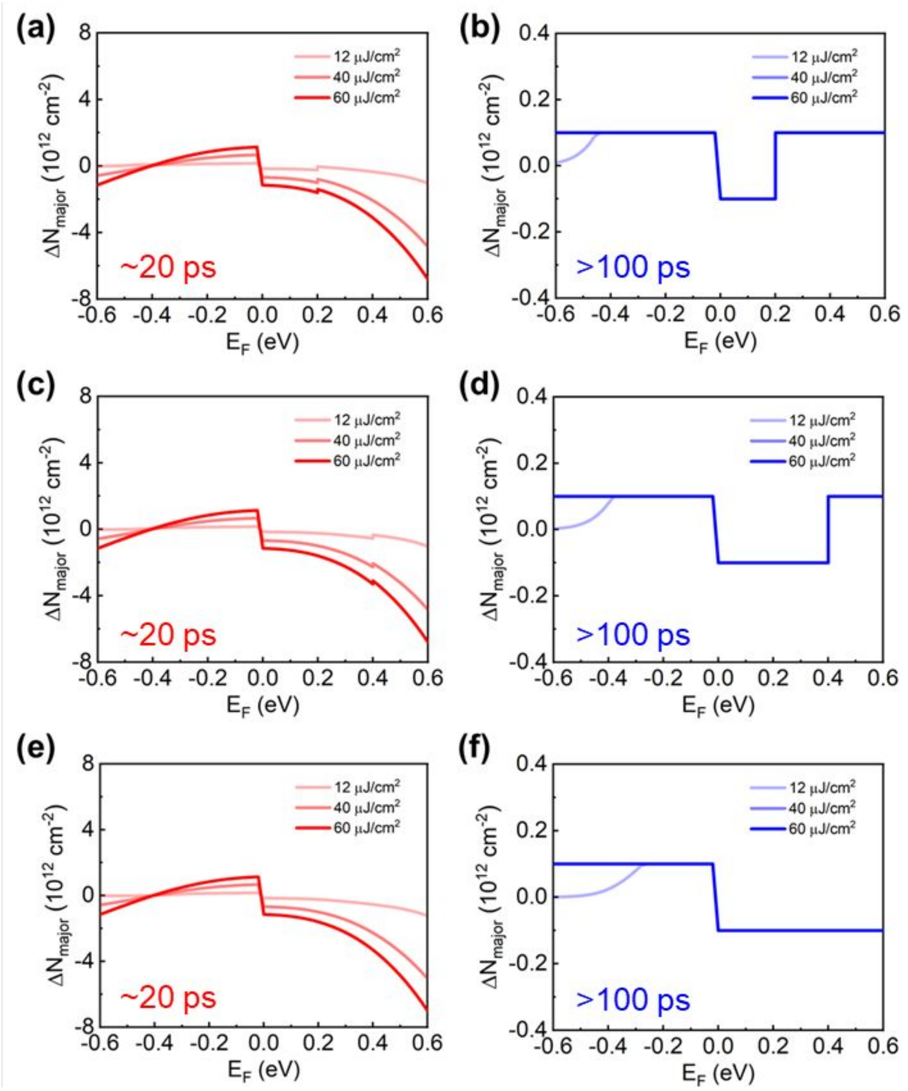

Figure S4 Majority carrier density change in graphene based on the defect-mediated PTE model involving one in-gap defect state with a fixed density and different energies. (a, c, e) Simulated E_F-dependent majority conducting carrier density change in graphene based on the PTE model involving one in-gap defect state with a fixed density (N_D = 1×10^{11} cm^{-2}) before the hot carriers injected in WS_2 are transferred back to graphene at different pump fluences: (a) E_D = 0.2 eV; (c) E_D = 0.4 eV; (e) E_D = 0.6 eV above the Dirac point. (b, d, f) Simulated E_F-dependent majority conducting carrier density change in graphene based on the PTE model involving one in-gap defect state with a fixed density (N_D = 1×10^{11} cm^{-2}) after the hot carriers injected in WS_2 are transferred back to graphene at different pump fluences: (b) E_D = 0.2 eV; (d) E_D = 0.4 eV; (f) E_D = 0.6 eV above the Dirac point.

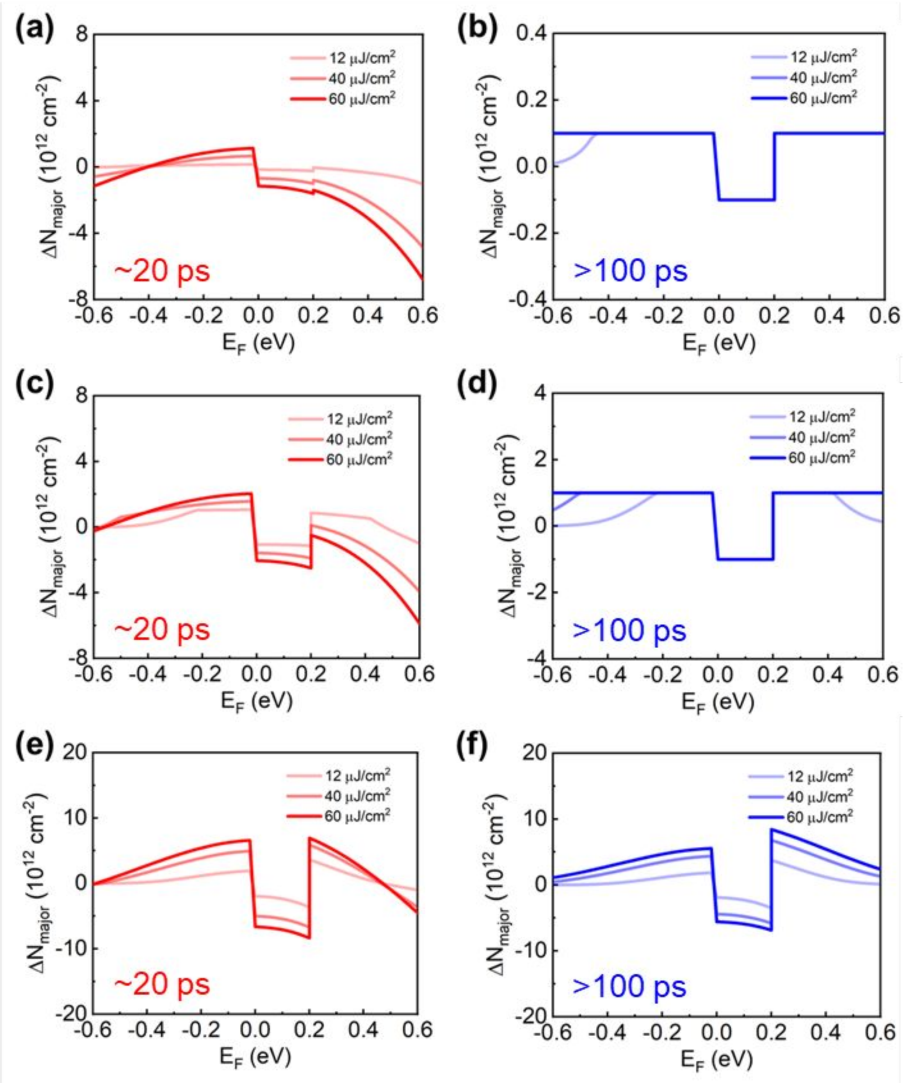

Figure S5 Majority carrier density change in graphene based on the defect-mediated PTE model involving one in-gap defect state with a fixed energy and different densities. (a, c, e) Simulated E_F-dependent majority conducting carrier density change in graphene based on the PTE model involving one in-gap defect state with a fixed energy (E_D = 0.2 eV) before the hot carriers injected in WS_2 are transferred back to graphene at different pump fluences: (a) N_D = 1×10^{11} cm^{-2}; (c) N_D = 1×10^{12} cm^{-2}; (e) N_D = 1×10^{13} cm^{-2}. (b, d, f) Simulated E_F-dependent majority conducting carrier density change in graphene based on the PTE model involving one in-gap defect state with a fixed density (N_D = 1×10^{11} cm^{-2}) after the hot carriers injected in WS_2 are transferred back to graphene at different pump fluences: (b) N_D = 1×10^{11} cm^{-2}; (d) N_D = 1×10^{12} cm^{-2}; (f) N_D = 1×10^{13} cm^{-2}.

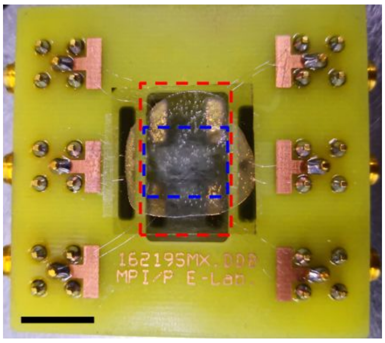

Figure S6 An optical image of the as-prepared electrochemically gated graphene-WS_2 heterostructure. The black scale bar in the lower left corner is 1 cm. The CVD-grown monolayer WS_2 film (dashed red region) is ~1 cm × 2 cm and the transferred CVD-grown monolayer graphene (dashed blue region) is ~1 cm × 1 cm. The electrodes consist of 5 nm Cr/50 nm Au and are connected to T-contacts by wire bonding. The two gate electrodes are ~2 mm × 15 mm, and the four electrodes for the four-point probe measurements are ~2 mm × 5 mm.

References

(1) Jnawali, G.; Rao, Y.; Beck, J. H.; Petrone, N.; Kymissis, I.; Hone, J.; Heinz, T. F. Observation of Ground- and Excited-State Charge Transfer at the C_{60}/Graphene Interface. *ACS Nano* **2015**, *9* (7), 7175–7185.

(2) Yan, J.; Zhang, Y.; Kim, P.; Pinczuk, A. Electric Field Effect Tuning of Electron-Phonon Coupling in Graphene. *Phys. Rev. Lett.* **2007**, *98* (16), 166802.

(3) Frenzel, A. J.; Lui, C. H.; Shin, Y. C.; Kong, J.; Gedik, N. Semiconducting-to-Metallic Photoconductivity Crossover and Temperature-Dependent Drude Weight in Graphene. *Phys. Rev. Lett.* **2014**, *113* (5), 56602.

(4) Nair, R. R.; Blake, P.; Grigorenko, A. N.; Novoselov, K. S.; Booth, T. J.; Stauber, T.; Peres, N. M. R.; Geim, A. K. Fine Structure Constant Defines Visual Transparency of Graphene. *Science* **2008**, *320* (5881), 1308.

(5) Tielrooij, K.-J.; Song, J. C. W.; Jensen, S. A.; Centeno, A.; Pesquera, A.; Elorza, A. Z.; Bonn, M.; Levitov, L. S.; Koppens, F. H. L. Photoexcitation Cascade and Multiple Hot-Carrier Generation in Graphene. *Nat. Phys.* **2013**, *9* (4), 248–252.

(6) Chen, Y.; Li, Y.; Zhao, Y.; Zhou, H.; Zhu, H. Highly Efficient Hot Electron Harvesting from Graphene before Electron-Hole Thermalization. *Sci. Adv.* **2019**, *5* (11), eaax9958.

(7) Aeschlimann, S.; Rossi, A.; Chávez-Cervantes, M.; Krause, R.; Arnoldi, B.; Stadtmüller, B.; Aeschlimann, M.; Forti, S.; Fabbri, F.; Coletti, C. Direct Evidence for Efficient Ultrafast Charge Separation in Epitaxial WS_2/Graphene Heterostructures. *Sci. Adv.* **2020**, *6* (20), eaay0761.

(8) Henck, H.; Aziza, Z. Ben; Pierucci, D.; Laourine, F.; Reale, F.; Palczynski, P.; Chaste, J.; Silly, M. G.; Bertran, F.; Le Fevre, P. Electronic Band Structure of Two-Dimensional WS_2/Graphene van Der Waals Heterostructures. *Phys. Rev. B* **2018**, *97* (15), 155421.

(9) Schuler, B.; Qiu, D. Y.; Refaely-Abramson, S.; Kastl, C.; Chen, C. T.; Barja, S.; Koch, R. J.; Ogletree, D. F.; Aloni, S.; Schwartzberg, A. M. Large Spin-Orbit Splitting of Deep in-Gap Defect States of Engineered Sulfur Vacancies in Monolayer WS_2. *Phys. Rev. Lett.* **2019**, *123* (7), 76801.

(10) Rosenberger, M. R.; Chuang, H.-J.; McCreary, K. M.; Li, C. H.; Jonker, B. T. Electrical Characterization of Discrete Defects and Impact of Defect Density on Photoluminescence in Monolayer WS_2. *ACS Nano* **2018**, *12* (2), 1793–1800.

(11) Edelberg, D.; Rhodes, D.; Kerelsky, A.; Kim, B.; Wang, J.; Zangiabadi, A.; Kim, C.; Abhinandan, A.; Ardelean, J.; Scully, M. Approaching the Intrinsic Limit in Transition Metal Diselenides via Point Defect Control. *Nano Lett.* **2019**, *19* (7), 4371–4379.
